# MMP7 expression regulated by endocrine therapy in ERβ-positive colon cancer cells

**DOI:** 10.1186/1756-9966-28-132

**Published:** 2009-09-29

**Authors:** Yu-Jing Fang, Zhi-Zhong Pan, Li-Ren Li, Zhen-Hai Lu, Li-Yi Zhang, De-Sen Wan

**Affiliations:** 1 Department of Colorectal Surgery, State Key Laboratory of Oncology in South China, Cancer Center, Sun Yat-sen University, Guangzhou, PR China; 2 Department of Experimental Research, State Key Laboratory of Oncology in South China, Cancer Center, Sun Yat-sen University, Guangzhou, PR China

## Abstract

**Background:**

Many studies have shown that colon cancer is an estrogen-dependent carcinoma. This study explored the efficacy of endocrine therapy in colon cancer cells with high metastatic potential (HT29). We investigated the proliferation of HT29 cells after exposure to endocrine therapy (tamoxifen) and 5-FU.

**Methods:**

Apoptosis was evaluated using flow cytometry. The expression of matrix metalloproteinases 7 (MMP-7) and estrogen receptor beta (ERβ) was measured by reverse transcription-polymerase chain reaction (RT-PCR) and western blot. The migration capability of treated cells was determined with wound scratch assay.

**Results:**

Tamoxifen alone, 5-FU alone, and the combination of the two drugs can significantly inhibit HT29 cell proliferation and migration, block the cells in G_2_/M phase and induce cell apoptosis. These drugs also can down-regulate MMP7 and ERβ expression.

**Conclusion:**

Our findings suggest that endocrine therapy is an efficient therapy for inhibiting ERβ-positive colon cancer cell proliferation and migration via down-regulation of MMP7.

## Background

Colorectal cancer is a growing health problem. In 2002 over one million new cases of colorectal cancer were diagnosed, and 529,000 people died from the disease, with the majority of deaths attributable to distant metastases [1]. The liver is a frequent site of colorectal metastases, and 15% to 25% of these patients have liver metastases at diagnosis [2]. About 50% to 60% of colorectal cancer patients will eventually develop advanced or metastatic disease [3]. Despite advances in survival with chemotherapy or surgical resection of hepatic metastases, the majority of patients still experience disease recurrence [4].

Many studies observed that the estrogen receptor beta (ERβ) is significantly related to cancer metastases [5-7]. Kuiper et al. first characterized ERβ in the rat prostate and ovary [8]. ERβ is the dominant receptor in human colonic mucosa, as many studies have shown that ERβ is more highly expressed than ERα in colon tissue [9-12]. Animal studies also revealed roles for ERβ in many tissues and organs, including the ovary, uterus, mammary gland, ventral prostate, salivary gland, immune system and central nervous system [13-17]. Currently, ERβ is the only ER identified in colon cell lines [10].

ERα and ERβ belong to a super-family of nuclear hormone receptors that function as transcription factors when they are bound to estrogens [18]. However, when selected ER modulators (SERMs), such as tamoxifen (TAM), bind to ERβ, they act as agonists rather than antagonists [19]. Additionally, Motylewska et al. showed that TAM exerted a very early and potent inhibitory effect on cancer cells, inducing total inhibition of cancer growth at a concentration of 10^-4 ^M [20].

Multiple factors, such as alterations in matrix metalloproteinases (MMPs), seem to be associated with polyp development. MMPs are a family of zinc-dependent [21,22] and calcium-dependent [22] endopeptidases that degrade matrix glycoproteins [21,23]. Eighteen types of MMPs, which play an important role in tumor invasion and metastases, have already been identified [24,25]. MMP7 (matrilysin) was first detected from the conditioned medium of a human rectal carcinoma cell line CaR-1 by Miyazaki et al. [26]. MMP7 is a target gene of the Wnt pathway, is an important biomarker of colorectal cancer ecurrence and metastases, and is overexpressed in malignant tumor and CRC liver metastases [27-29]. Gorodeski reported that estrogen may decrease the activation of MMP7 in vivo [30]. Thus, endocrine therapy may play a role in treating hormone-dependent cancers by decreasing the metastases that are caused by MMP7 activation. To test this hypothesis, we examined the ability of TAM to decrease MMP7 activation in the ERβ-positive colon cancer cell line HT29.

## Methods

### Cell culture and treatment

HT-29 cells are highly metastatic colon carcinoma cells that were obtained from the American Type Culture Collection, Rockville, MD, USA. Cells were maintained in Dulbecco's modified Eagle medium supplemented with 10% fetal calf serum at 37°C in a humidified atmosphere of 5% CO_2_.

### Drug administration schedules

TAM and fluorouracil (5-FU) were purchased from Sigma (St Louis, MO). The drug-exposure schedules, which are summarized in Table 1, were as follows: (a) no treatment; (b) TAM alone (1 × 10^-7^, 1 × 10^-6^, 1 × 10^-5^, or 1 × 10^-4 ^M) for 48 h; (c) 5-FU alone (6.25, 12.5, 25, or 50 μM) for 72 h; (d) 12.5 μM 5-FU for 24 h followed by 12.5 μM 5-FU plus indicated TAM for 48 h. The experiments were performed in triplicate for each time point, and the means ± SD were calculated. Appropriate amounts of drug solution were added directly to the growth medium the day after plating. Control cells were plated in growth medium supplemented with 0.1% DMSO.

**Table 1:** Schedule of each group of treatment for three different times

**Group**	**24 h**	**48 h**	**72 h**
(a)	no treatment		
(b)	TAM	TAM	
(c)	5-FU	5-FU	5-FU
(d)	5-FU	5-FU+TAM	5-FU+TAM

### Drug sensitivity, as indicated by the MTT assay

To induce cell death, cells were treated with either TAM (Sigma, Cat. No. T-9262) dissolved in DMSO or 5-FU. The final concentrations ranged from 1 × 10^-7 ^to 1 × 10^-4 ^M for TAM and from 6.25 to 50 μM for 5-FU. To test the cytotoxicity of each drug, HT-29 cells in the exponential growth phase were seeded into 96-well cell plates in 100 μl of culture medium for 24 h prior to drug exposure and then treated with various concentrations of TAM, 5-FU, or a combination of these drugs. Cytotoxicity was evaluated using a tetrazolium-based semi-automated colorimetric (MTT) assay, with an ELISA reader at OD_490_.

### Flow cytometry analysis

HT-29 cells were seeded in 6-well plates at a density of 4 × 10^6 ^cell/well. Cells were treated with various concentrations of each drug for the appropriate times, incubated at 37°C, fixed in 70% ethanol, and labeled with propidium iodide solution (50 μg/ml; Sigma-Aldrich). The DNA content and cell cycle distribution of approximately 1 × 10^6 ^stained cells were analyzed using a FACScan flow cytometer (Becton Dickinson).

### Reverse transcriptase-polymerase chain reaction (RT-PCR)

Total RNA was isolated from 4 × 10^6 ^cells by TRIzol (Invitrogen, Carlsbad, CA, USA) according to the manufacturer's instructions. RNA was reverse transcribed in a total volume of 20 μl containing 2 μg RNA, 0.5 μg olig (dT)_15_, and 15 μl DEPC-treated water. Reverse transcription reaction was incubated at 30°C for 10 min, 48°C for 30 min, and 99°C for 5 min. After RT, the product was used to amplify MMP7, ERβ and normalized based on β-actin cDNA. PCR was performed using cDNA PCR kits (Takara, Cat. DRR019A, Japan) in a final volume of 50 μl according to the manufacturer's instructions. Amplification conditions were performed for 30 cycles (denaturation at 94°C for 1 min, annealing at 54°C for 1 min, and extension at 72°C for 1 min). The MMP7 primers were 5'-AGA TGT GGA GTG CCA GAT GT-3' (forward) and 5'-TAG ACT GCT ACC ATC CGT CC-3' (reverse). The ERβ primers were 5'-TGC TTT GGT TTG GGT GAT TGC-3' (forward) and 5'-TTT GCT TTT ACT GTC CTC TGC-3' (reverse). The β-actin primers were 5'-CGG GAC CTG ACT GAC TAC CTC A-3' (forward) and 5'-TCA AGA AAG GGT GTA ACG CAA CTA-3' (reverse). The PCR products were separated by electrophoresis on a 2% agarose gel and visualized by ethidium bromide staining and UV illumination. The expected sizes of the amplification products were 365 base pairs (bp) for MMP7, 259 bp for ERβ, and 656 bp for β-actin.

### Western blotting

HT-29 cells were exposed to TAM, 5-FU, or their combinations for various time points in various administration sequences. After treatment, 5 × 10^6 ^cells were collected for protein extraction. Cell pellets were washed in PBS twice and then lysed in 80 μl lysis buffer (0.1% SDS, 50 mmol/L Tris·HCl pH 8.0, 150 mmol/L NaCl, 1 mmol/L EDTA, 100 μg/ml PMSF, 1 μg/ml Aprotinin, 1% NP-40) for 30 min on ice. After centrifugation at 12,000 rpm for 5 min at 4°C, the supernatants were collected and frozen at -80°C until analysis. Forty micrograms of total protein were loaded in each well of a 10% SDS-PAGE gel. Proteins were transferred to Hybond P polyvinylidene fluoride membranes (Amersham Pharmacia Biotech, Amersham, UK), which were then blocked in 5% dried skimmed milk powder in TBST (Tween 20/TBS) for 3 h at room temperature. Membranes were probed with primary antibodies (mouse monoclonal MMP7 and ERβ antibody, 1/1000) and then horseradish peroxidase-conjungated second antibody. After washing, the immunoreactive protein was detected using chemiluminescence (Cell Signaling).

### Wound scratch assay

HT29 cells (2 × 10^5^) were cultured to confluent cell monolayers in medium containing 10% FBS on 6-well tissue culture dishes. Cells were carefully wounded using sterile 20-μl pipette tips. The wounded monolayers were washed twice with PBS to remove nonadherent cells and incubated at 37°C in complete media. The cells were then incubated in TAM (according to the drug administration schedule) for 24 h, 48 h, or 72 h. The wound edges were imaged by phase-contrast microscopy, and the extent of migration was analyzed using the NIH image software .

### Statistical analysis

The results are presented as the mean ± SD. *P *values less than 0.05 were considered statistically significant.

## Results and Discussions

### Effects of each treatment on proliferation and apoptosis of HT29 cells

**Figure 1:**
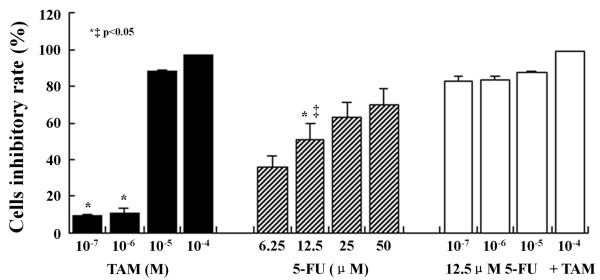
**Cytotoxic effect of TAM, 5-FU or a combination of these two drugs on HT29 cells**. Each point is the mean ± SD of three separate experiments. *P < 0.05, TAM vs. 12.5 μM 5-FU; ‡ P < 0.05, 12.5 μM 5-FU vs. 12.5 μM 5-FU+TAM.

Proliferation of HT29 cells was not inhibited by the lower doses (10^-7 ^and 10^-6 ^M) of TAM at 24 and 48 h but was significantly inhibited in a dose- and time-dependent manner by the higher doses (10^-5 ^and 10^-4 ^M) (Figure 1). On the contrary, HT29 cells were significantly affected by 5-FU in the range of concentrations between 6.25 and 50 μM at 72 h. Because of the strong effect of 5-FU, we choose the lower dose of 5-FU (12.5 μM) combined with the various doses of TAM (10^-7^, 10^-6^, 10^-5 ^and 10^-4 ^M) to treat the cells. Using 12.5 μM 5-FU in combination with TAM showed significant inhibition of the rate of HT29 cell proliferation compared to single treatments (Figure 1).

We analyzed the cell cycle distribution after drug treatment and found evidence of a preferential block of colon cancer cells in the G_2_/M phase. In cells treated with TAM, when the drug concentration is increased from 10^-7 ^to 10^-4 ^M, the percentage of cells in the G_2_/M phase decreased from approximately 9.1 to 2.4% and the percentage of cells in the G_0_/G_1 _phase decreased from 75.9 to 30%. Increasing the dose of 5-FU, resulted in a growth arrest at S phase, and the colon cancer cells were completely blocked in G_2_/M phase. When 12.5 μM 5-FU was combined with increasing doses of TAM, a decreased percentage of cells was detected in G_0_/G_1 _phase, and cells were completely blocked in G_2_/M phase (Table 2).

**Table 2:** Effects of each drug on cell cycle in HT29 cell

**Group**		**G1 (%)**	**G2/M (%)**	**S (%)**
Control		82.2 ± 5.4	2.2 ± 0.5	15.5 ± 1.8
TAM (M)	10^-7^	75.9 ± 5.7	9.1 ± 2.1	15 ± 2.5
	10^-6^	75.8 ± 4.5	9.2 ± 1.9	15 ± 2.1
	10^-5^	63.2 ± 5.1	7.3 ± 1.4	29.5 ± 3.4
	10^-4^	30 ± 5.6	2.4 ± 0.6	67.6 ± 4.5
5-FU (μM)	6.25	66.7 ± 5.4	0	33.3 ± 3.8
	12.5	71.1 ± 6.2	0	28.9 ± 4.2
	25	73.7 ± 7.4	0	26.3 ± 3.2
	50	79.8 ± 7.7	0	20.2 ± 3.1
12.5 μM 5-FU +TAM (M)	10^-7^	75.0 ± 8.1	0	25.0 ± 4.2
	10^-6^	67.8 ± 6.3	0	32.2 ± 3.1
	10^-5^	51.8 ± 5.5	0	48.2 ± 4.7

Each value is the mean ± SD of three separate experiments.

**Figure 2:**
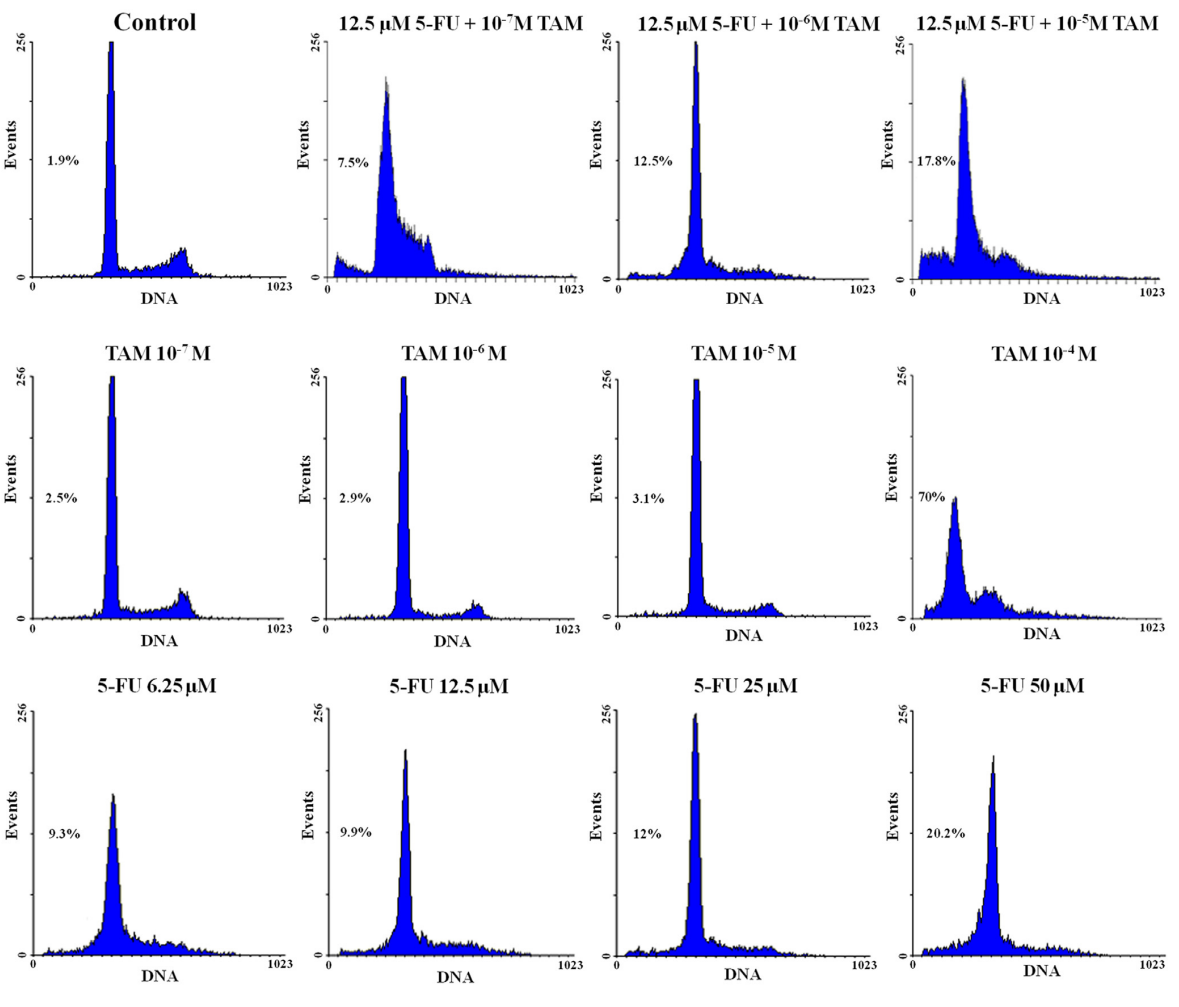
**Effect of each drug on apoptosis in HT29 colon cancer cells**. The ratio of apoptotic cells increased in dose-dependent manner which were measured in HT29 cells treated with combined drugs (12.5 μM 5-FU with each dose of TAM), TAM and 5-FU, respectively.

Flow cytometry analysis confirmed the apoptosis rates of HT29 cells under each treatment. Based on the DNA histograms, 2.5, 2.9, 3.1 and 69.9% of the cells treated with 1 × 10^-7^, 1 × 10^-6^, 1 × 10^-5 ^and 1 × 10^-4 ^M TAM for 48 h were in sub-G1 phase. Among cells treated with increasing doses (6.25-50 μM) of 5-FU for 72 h, 9.3, 9.9, 12 and 20.2% of cells were in sub-G1 phase. When the two drugs were combined (12.5 μM 5-FU with each dose of TAM) for 72 h, 7.5, 12.5, and 17.8% of cells were in sub-G1 phase, These differences were significantly increased compared to control HT29 colon cancer cells (1.9%) (Figure 2).

### Migration capability of colon cancer cells treated TAM alone and in combination with 5-FU

**Figure 3:**
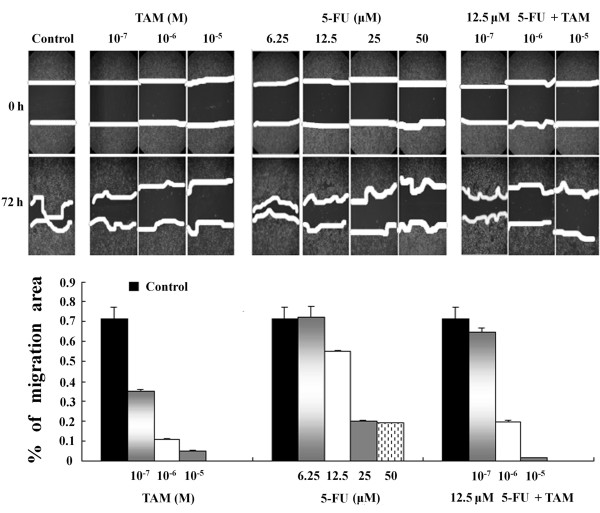
**Cell migration of HT29 colon cancer cells over a 72-h period in response to different drugs after compared as determined with the wound scratch assay (values are mean ± SD of three independent experiments)**.

The ability of tumor cells to migrate is closely associated with their metastatic potential, and the wound scratch assay was conducted to measure this potential. The percentage of migration area covered after 72 h was 71.6 ± 5.9% for control cells; 34.9 ± 1%, 11.1 ± 0.4% and 4.9 ± 0.4% for cells treated with TAM (10^-7^, 10^-6 ^and 10^-5 ^M, respectively); and 55 ± 0.4%, 20.1 ± 0.2% and 18.8 ± 0.4% for cells treated with 5-FU (12.5, 25 and 50 μM, respectively). The percentage of migration area of the drug treatments was significantly lower than that of the control cells (P < 0.0001). Based on the above results, the lower dose of 5-FU (12.5 μM) was combined with each dose of TAM (10^-7^, 10^-6 ^and 10^-5 ^M) for further assays. The percentage of migration area for the combined treatment was 65 ± 2%, 19.5 ± 1% and 1.4 ± 0.2% at 10^-7^, 10^-6 ^and 10^-5 ^M TAM, respectively (Figure 3). The anti-metastatic effect of TAM on HT29 cells was confirmed to be dose-dependent, and it was co-effect with 5-FU at higher dose as well. As the wound gap is dismissed (because of cell death) in the cells under the treatment of 10^-4^M TAM and the almost same results of control group and 6.25 μM 5-FU, so we discard the results of these two concentrations in this part.

### Effects of TAM and 5-FU on MMP7 and ERβ mRNA expression

**Figure 4:**
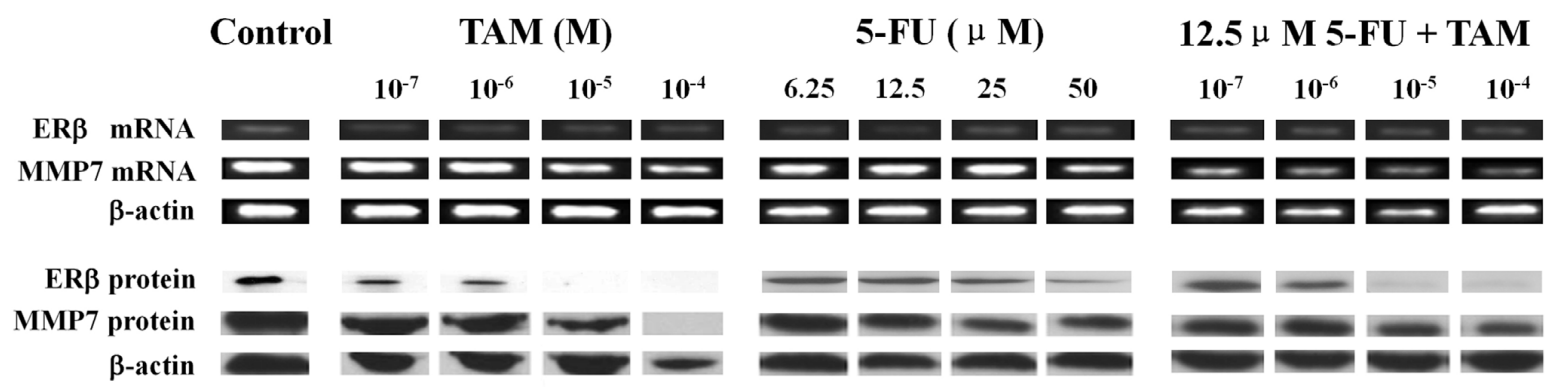
**Down-regulation of MMP7 and ERβ levels in HT29 cells, following treatment with TAM, 5-FU or 12.5 μM 5-FU combined with indicated concentrations of TAM**.

We were interested in determining whether the MMP7 and ERβ genes could be inhibited by TAM and 5-FU. We performed RT-PCR with MMP7 and ERβ primers on cDNA that was reverse transcribed from RNA isolated from HT29 cells. As expected, MMP7 mRNA was down-regulated in a concentration-dependent manner after incubation with different concentrations of TAM, 5-FU, and the combination of these two drugs. However, the ERβ mRNA was not significantly altered by the treatments (Figure 4).

### Effect of TAM alone and combined with 5-FU on MMP7 and ERβ protein expression in HT29 cells

We confirmed that HT29 cells express ERβ but do not express ERα (data not shown). TAM (10^-4 ^and 10^-5 ^M) down-regulated MMP7 and ERβ protein levels after 48 h. Treatment of HT29 cells with 5-FU (0, 6.25, 12.5, 25, 50 μM) for 72 h showed a trend of diminished expression of MMP7, but it significantly down-regulated ERβ protein levels only when given at 50 μM. The combination treatment of 12.5 μM 5-FU and each dose of TAM significantly diminished expression of MMP7 and ERβ. Additionally ERβ protein level was completely down-regulated in response to 12.5 μM 5-FU plus 10^-5 ^M TAM (Figure 4).

In this paper, we present two important findings. First, SERMs such as TAM alone and in combination with 5-FU can act as a chemo-endocrine therapy that efficiently inhibits the proliferation and induces the apoptosis of ERβ-positive colon cancer cells. Second, TAM alone and in combination with 5-FU can effectively inhibit the migration of ERβ-positive colon cancer cells by down-regulating MMP7 and ERβ expression. To determine whether TAM can inhibit ERβ and MMP7 transcription in colon cancer cells, an ERβ-positive colon cancer cell line HT29 was treated by TAM alone and in combination with 5-FU. As shown in Figure 4, ERβ and MMP7 were present in HT29 cells and were inhibited following TAM and 5-FU treatment. These genes were especially down-regulated by the treatment of TAM and 5-FU together.

TAM is an antiestrogenic compound with a pure ERα selective partial agonist/antagonist activity and a pure β selective antagonist activity. These effects result in the down-regulation of ERs. It is the first drug in the class of SERMs [31-33]. Several SERMs are currently in various stages of clinical testing. A recent study by Motylewska et al[20] indicates that TAM and estradiol inhibit colon cancer growth and increase the cytotoxic effect of FU. This study confirmed the importance of hormone steroids in colon carcinogenesis and even suggested new therapeutic schemes.

Endocrine therapy of colorectal carcinoma has been suggested for decades, and there is some evidence to support its use on colon cancer. Epidemiological data and gender differences in the incidence of colon cancer suggest that colon cancer is a hormone-dependent cancer. ERβ was identified and is the predominant ER in colon tissue [12], and overexpression of ERβ in the human colon, coupled with negligible expression of ERα, suggests that ERβ is involved in the protective effect of endocrine therapy on colonic carcinogenesis. In addition, ERβ inhibits tumor cell invasion and migration [6]. Based on the above evidence, we tested cell migration in response to the different drug treatments by cell scratching assay. Our results support the hypothesis that ERβ-positive cell migration can be inhibited by endocrine therapy.

Our data clearly demonstrated that MMP7 was down-regulated by TAM, which induces apoptosis through ERβ. Some researchers have reported that ERβ induces apoptosis in colon cancer Lovo cells due to increased p53 signaling and have proposed that a reduction in β-catenin protein is the cause of inhibition of cell proliferation [34]. MMP7 overexpression is an early event in the carcinogenetic cascade as normal colonic mucosa progresses to adenoma [35]. β-catenin, bound to T cell factor in the cytoplasm, enters the nucleus and promotes the expression of target genes including cyclo-oxygenese, c-myc and MMP7. These proteins are overexpressed in colorectal cancer, and a positive correlation has been demonstrated between nuclear β-catenin protein levels and MMP7 transcription in colorectal cancer [36]. Others have also demonstrated that MMP7 protein and mRNA are consistently expressed in liver metastases [29,37]. Thus, we suggest MMP7 as a therapeutic target for endocrine therapy of colorectal carcinoma.

## Conclusion

The results support endocrine therapy as an efficient therapy for colon cancer cells. Additionally, chemo-endocrine therapy can also effectively down-regulate MMP7, which in turn can influence tumor cell invasion and migration. Further morphological studies in ER knockout models should clarify the role of ERβ in colon tissue and confirm the results from our cytology studies.

## Abbreviations

ERβ: Estrogen Receptor Beta; 5-FU: Fluorouracil; MMPs: Matrix Metalloproteinases; MMP-7: Matrix Metalloproteinases 7; RT-PCR: Reverse Transcription Polymerase Chain Reaction; SERMs: Selected ER Modulators; TAM: Tamoxifen.

## Competing interests

The authors declare that they have no competing interests.

## Authors' contributions

YJF participated in the study design, performed the data analysis, draft the manuscript. PZZ, LLR and LZH participated in the study design and helped draft the manuscript. ZLY performed the experiments. WDS was responsible for the overall study design. All authors read and approved the final manuscript.
